# Enhancing Quality of Life in Patients With Hypothyroidism Using a Scientific Yoga Module: Randomized Controlled Trial

**DOI:** 10.2196/54078

**Published:** 2025-06-26

**Authors:** Savithri Nilkantham, Amit Singh, Vijaya Majumdar, Harini K N, Snigdha Atmakur

**Affiliations:** 1 Department of Yoga & Life Sciences S-VYASA Yoga University Swami Vivekananda Yoga Anusandhana Samsthana Bengaluru India; 2 Department of Strategy and General Management T A Pai Management Institute Manipal Academy of Higher Education Manipal India

**Keywords:** hypothyroidism, tele-yoga, digital health, scientific yoga module, health-related-quality of life, randomized controlled trial

## Abstract

**Background:**

The impact of hypothyroidism on quality of life is extensively documented, highlighting its substantial physical, psychological, and social burden. Yoga has demonstrated promising therapeutic benefits in improving hypothyroidism outcomes. Leveraging telehealth’s growth, this study used a rigorously designed scientific yoga module specifically tailored for digital delivery for patients with hypothyroidism undergoing levothyroxine treatment.

**Objective:**

This study aimed to assess the impact of a 6-month tele-yoga intervention in patients with hypothyroidism by comparing outcomes between those receiving levothyroxine combined with tele-yoga and those receiving only levothyroxine treatment.

**Methods:**

A single-blinded, 2-arm, parallel-group randomized controlled trial was conducted for 6 months (April 1, 2022-September 30, 2022) with 134 clinically diagnosed patients with hypothyroidism recruited from the Arogyadhama Holistic Health Home registry (2013-2021). Participants were randomized to either a yoga intervention group or a waitlist control group with 67 in each group and assessed at 3 time points (before, in the middle of, and after the intervention) for primary and secondary outcomes. The 36-Item Short Form Health Survey for health-related quality of life was used as a primary measure, whereas secondary measures included thyroid profile, BMI, blood pressure, the Fatigue Assessment Scale, the Perceived Stress Scale, and the Gita Inventory of Personality. Clinical data were collected via online questionnaires, and laboratory data (thyroid profile blood pressure and anthropometric measurements) were obtained in person using standardized instruments. A generalized linear model with repeated-measure ANOVA was used to evaluate both within- and between-group effects. In addition, in the yoga intervention group, performance was assessed using a yoga performance assessment scale, and satisfaction was measured through a structured feedback survey.

**Results:**

The intervention showed highly significant effects across all domains of the primary outcome measure (*P*<.001), with the most notable effects on mental health (*F*_2, 118_=425.88; η^2^=0.88), energy and vitality (*F*_2, 118_=371.73; η^2^=0.86), and role limitations—emotional (*F*_2, 118_=335.45; η^2^=0.85). Secondary measures also showed significant improvements (*P*<.001), except for thyroxine (*P*<.014). Average yoga performance assessment scores increased significantly from 65.08 (SD 10.97) to 88.62 (SD 11.18; *P*<.001), indicating that most participants could easily perform the practices. Overall, 95% (64/67) of the participants in the yoga intervention group expressed high satisfaction with the tele-yoga intervention.

**Conclusions:**

This clinical trial is the first to demonstrate the benefits of a digitally delivered scientific yoga module combined with levothyroxine treatment for hypothyroidism. It highlights the efficacy of instructor-led tele-yoga as a scalable eHealth intervention, enhancing accessibility, long-term engagement, and sustainable health outcomes. Patients receiving tele-yoga alongside levothyroxine showed significantly greater improvements than those on levothyroxine alone, highlighting the value of integrating eHealth into thyroid care for a more comprehensive, patient-centered approach.

**Trial Registration:**

Clinical Trials Registry–India CTRI/2022/03/041047; https://ctri.nic.in/Clinicaltrials/pmaindet2.php?EncHid=NjY5NzI

## Introduction

### Background

Hypothyroidism, characterized by underactive thyroid gland function, is becoming increasingly prevalent, affecting up to 5% of the general population [1], with a further estimated 5% being undiagnosed in low-, middle-, and high-income nations [2]. The key determinants for this condition are environmental factors such as lifestyle, diet, iodine uptake, genetic susceptibility, use of new endocrine-disrupting chemicals, and the immune status of an individual [3]. It has emerged as a potential contributor to comorbidities, increasing the risk of cardiovascular and neuropsychiatric diseases [4]. Furthermore, hypothyroidism is accentuated by risk factors such as stress, depression, obesity, increase in triglycerides, hyperglycemia, hypertension, and others, impacting the overall health and quality of life (QoL).

Clinical and experimental studies indicate that serum thyroxine and triiodothyronine are synthesized by thyrotrope cells in the anterior pituitary gland and are regulated by the thyroid-stimulating hormone (TSH), which in turn controls the endocrine function of the thyroid gland [5]. Furthermore, thyroid hormone metabolic activity takes place in the peripheral tissues, especially in the liver with the conversion of thyroxine to triiodothyronine to maintain thyroid gland functions [6]. In hypothyroidism, due to a lack of serum thyroxine secretion, bodily functions are affected, which manifests in serious complications. As per the American Association of Clinical Endocrinology medical guidelines, in hypothyroidism, TSH levels are elevated (≥10 mIU/L), in contrast to a reduction in the levels of free thyroxine (≤1.0-1.7 ng/dL) and free triiodothyronine (≤2.1-4.1 pg/mL) [7]. Hence, the TSH screening test is considered essential for thyroid dysfunction, and a disproportionately high concentration of TSH may warrant thyroxine and triiodothyronine measurements as well [8-10].

A recent meta-analysis that included 17 studies with 1163 patients with hypothyroidism concluded that the direct influence of hypothyroidism on the autonomic nervous system negatively affects a person’s health by causing fluctuations in BMI, blood pressure (BP), and mental states [11]. Although the biochemical target is achieved using levothyroxine as a conventional medication, studies have reported that approximately 5% to 10% of the patients still have complaints due to persistent symptoms such as lack of energy, increase in stress, excessive fatigue, cognitive problems, musculoskeletal pain, and weight gain, impacting the QoL [12,13]. Often, patients are left with no choice but to depend on levothyroxine medication alone as a standard of care, leading to not only notable medical costs but also an everlasting struggle with symptoms [14].

### Tele-Yoga and Telehealth Focus

The National Institutes of Health, a part of the US Department of Health and Human Services, recognizes yoga as a complementary mind-body therapy for thyroid disorders [15,16]. Yoga exerts therapeutic effects on various organ functions, including the endocrine system, with numerous studies evaluating its effectiveness for hypothyroidism [17-20]. The Ministry of Ayush (which stands for ayurveda, yoga and naturopathy, Unani, Siddha, and homeopathy) under the Government of India issued an advisory recommending the use of digital platforms for yoga therapy [21], which included yoga practices such as postures (*asanas*), breathing techniques (*pranayama*), meditation (*dhyana*), and counseling through internet-based video platforms to facilitate widespread access to yoga practices [21]. This enabled a broad selection of sessions tailored to the skill levels, styles, and health conditions of participants, facilitating personalized practices tailored to individual goals and wellness objectives; addressing diverse schedules, time limitations, and mobility challenges; providing a cost-effective alternative to in-person classes with budget-friendly options; and enhancing the capacity, quality, and efficiency of health care resource allocation and extending the reach of health care services worldwide [22-25].

### Aim and Objectives

This study aimed to assess the impact of a 6-month digitally delivered scientific yoga module intervention on patients with hypothyroidism, comparing outcomes between levothyroxine treatment combined with the scientific yoga module and levothyroxine treatment alone. The objective was to compare the effectiveness of intervention between the yoga intervention group and the waitlist control group by testing the hypothesis on the primary measure of the 36-Item Short Form Health Survey (SF-36) for health-related QoL and the secondary measures of thyroid hormone levels (thyroxine, triiodothyronine, and TSH), BMI, BP, the Perceived Stress Scale (PSS), the Fatigue Assessment Scale (FAS), and the Gita Inventory of Personality (GIP). The findings of this study will provide valuable insights for advancing integrative medicine practices that leverage tele-yoga eHealth solutions to improve health care quality and accessibility.

## Methods

**Figure 1:**
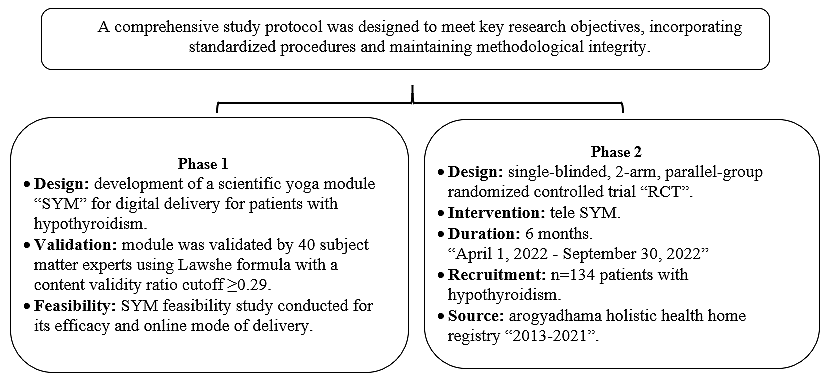
Development of the tele-yoga study protocol. RCT: randomized controlled trial; SYM: scientific yoga module.

This randomized controlled trial (RCT) is the second phase of a rigorously structured and published protocol [26] and adheres to standardized procedures developed in this protocol to meet key research objectives and maintain methodological integrity throughout the study, as shown in Figure 1.

### Phase 1 of the Protocol: Development, Validation, and Feasibility of a Scientific Yoga Module

In the first phase of the protocol, a thoroughly validated and reliable scientific yoga module designed for digital delivery was developed following an extensive literature review [26]. The researchers initially proposed 31 yoga practices, of which 24 were endorsed by 40 subject matter experts in integrative medicine. This selection was based on a content validity ratio analysis, applying the Lawshe formula with a threshold of ≥0.29, resulting in integrating these practices into the tele-scientific yoga module framework. Tailored specifically for patients with hypothyroidism on levothyroxine medication, this module was tested for feasibility and optimized for internet-based delivery. This study’s findings were published, indicating that the tele-yoga intervention was safe and effective as a supplementary treatment for hypothyroidism alongside the standard of care [27].

### Phase 2 of the Protocol: RCT

The study reported in this paper represents the second phase of the research protocol, during which the researchers conducted a 6-month RCT using tele-yoga as an intervention for 134 patients with hypothyroidism. The methodology and study details are outlined in this section.

#### Design of the RCT

This study was a single-blinded, 2-arm, parallel-group trial and followed the guidelines of the CONSORT (Consolidated Standards of Reporting Trials) 2017 Statement for Randomized Trials of Nonpharmacologic Treatments, as well as its extension [28,29].

#### Inclusion and Exclusion Criteria

Participants comprised both male and female patients eligible for inclusion if they were aged between 18 and 60 years with clinically confirmed primary hypothyroid disease; had elevated TSH levels of >5 mIU/L and low thyroxine levels of <4.5 μg/dL, and were on a stable dose of levothyroxine for at least 6 months before enrollment in the trial. The researchers further reviewed the records to identify patients with controlled comorbidities, which included a 9-item Patient Health Questionnaire score of ≥10 (score range 0-27; higher scores indicate more severe depressive symptoms), at least one poorly controlled cardiometabolic parameter (glycated hemoglobin of ≥8%, systolic BP of ≥140 mm Hg, or low-density lipoprotein cholesterol of ≥130 mg/dL), and BMI of ≤40; patients who had not engaged in general yoga for a minimum of 3 months before the study and had not planned to initiate practice during the trial period; and patients with access to the internet and who had a smartphone, tablet, laptop, or television for joining the live tele-yoga trial.

The selection process excluded certain patients to ensure the study’s validity, safety, and feasibility. Exclusions included those with thyroid malignancy, secondary hypothyroidism, or other autoimmune disorders (eg, lupus or multiple sclerosis) to minimize confounding factors; those with severe psychiatric disorders (eg, schizophrenia, mania, or dissociative disorder) due to potential adherence challenges; those on medications such as lithium that impair thyroid function; individuals with significant head or neck trauma, surgery, or radiotherapy as this could affect thyroid function; pregnant women or those planning to conceive due to potential impacts on thyroid function and yoga safety; and patients with recent surgery (within 6 months), hospitalization for major illness (within 4 weeks), or visual or hearing disabilities to ensure stable health and effective participation in the internet-based yoga sessions.

#### Sample Size Calculation

The estimation of the sample size was calculated based on effect size, statistical power of the study, feasibility, precision about the mean, and variance using the G*Power software [30]. Furthermore, we computed the sample size for this trial based on earlier study involving yoga and QoL measures [31]. With an effect size of 0.8, significance fixed at .05, the power of the study at 0.95, and an attrition rate of 25%, a total sample size of 120 participants was calculated for this study. This sample size was specifically chosen to ensure robust statistical power, providing adequate sensitivity to detect meaningful effects and ensuring the validity and reliability of the study outcomes.

#### Recruitment Process

The study participants were recruited from the registered patient directory of the Arogyadhama Holistic Health Home (AHHH), a residential integrative medical hospital located within a major industrial and employment hub in Bengaluru, Karnataka, India. AHHH specializes in yoga and naturopathy and treats patients with its unique system of therapeutic healing of the disease including common conditions such as thyroid disorders, diabetes, hypertension, arthritis, allergies, anxiety, stress, and more. It is a multidisciplinary hospital with >350 beds and an outpatient department providing attention, care, and treatment at any point in time for >500 patients with various ailments. A total of 707 patients diagnosed with hypothyroidism from the AHHH endocrinology department patient registry (January 2013 to December 2021) were approached. The screening and recruitment process for the study based on predefined inclusion and exclusion criteria commenced in January 2022 for a 6-month trial scheduled to run from April 1, 2022, to September 30, 2022. Information about the trial was disseminated to potential participants through posters and flyers shared via email and WhatsApp, with instructions to contact the study team using the provided details. Individuals expressing interest were sent a detailed screening questionnaire via Google Forms to collect data on sociodemographic characteristics, lifestyle habits, medical history, current health status, and other study-relevant factors. Eligible participants were then provided with a consent form to complete and return via email. All consented participants were randomized into 2 groups (the yoga intervention group and waitlist control group) using a computerized random number sequence [32] with an allocation ratio of 1:1 by an independent data researcher who had no role in this study [33]. Blinding of the participants was not possible in this trial; however, 2 data researchers in charge of data collection, preservation, and analysis were successfully blinded.

#### Yoga Intervention Group

##### Overview

**Figure 2:**
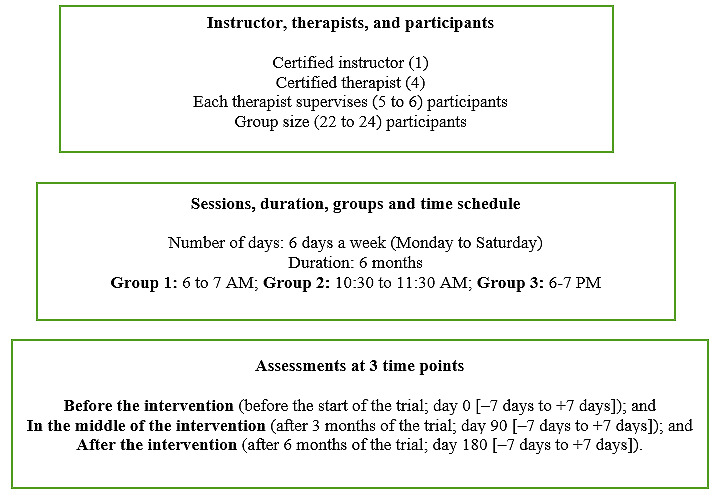
Tele–scientific yoga module intervention (session flowchart).

The participants in the yoga intervention group received scientific yoga module via a telehealth platform, whereby they were connected in real time through the internet and participated in the live-streamed yoga sessions. The sessions were conducted for 1 hour daily, 6 days a week for 6 months by a certified yoga instructor from the AHHH hospital with >10 years of teaching experience (including 5 years of teaching online). In total, 3 daily session slots were scheduled for early morning (6-7 AM), midmorning (10:30-11:30 AM), and evening (6-7 PM), and 3 groups were formed for the participants to attend their corresponding fixed time slots. As per tele-yoga guidelines, 1 session can accommodate no more than 25 participants and requires certified therapist supervision in the ratio of 1:5 (ie, 1 therapist for every 5 participants) to monitor the correctness of the practices. Hence, in addition to the certified instructor, 4 experienced yoga therapists from the AHHH hospital were recruited to ensure close participant supervision, with each therapist being allotted 5 to 6 fixed participants for a group size of approximately 22 to 24 participants, as shown in Figure 2.

The sessions were live-streamed by the certified instructor via a videotelephony software program—Microsoft Teams 365 (a widely used program for online yoga sessions with easy access) on a tablet. For the 3 separate groups, links were created by the certified instructor and shared with the certified therapists to join and supervise their assigned participants across the sessions. Each participant received their respective link as per their group timing and joined the tele-yoga sessions with ease. The attendance was downloaded from the computer application log sheet, and dropouts were monitored during the 6 months of the intervention. To compensate for missed sessions due to poor internet connectivity or personal reasons, participants received a step-by-step video of the module presented by the certified instructor [34] along with picture-based instructions for home practice at their convenience (Multimedia Appendix 1). The home practices were verified via submitted photos or videos and recorded in a separate home attendance log. In addition, for educational purposes, participants were provided with a guide via email explaining the disease, its causes, diagnosis, prognosis, and the benefits of the yoga module.

##### Orientation Session

A 1-week introductory session was conducted by yoga research experts from the AHHH hospital in a group format online using Microsoft Teams, allowing participants to engage collectively and interact with yoga experts, the certified instructor, and therapists. The sessions were held once daily in the mornings from 9 AM to 10:30 AM for 7 days, with each session lasting approximately 90 minutes and designed to achieve several objectives, including explaining the pathophysiology of the condition, introducing the certified instructor and therapists for the trial, familiarizing participants with the yoga module, organizing participants into groups, and providing detailed guidance on the use of group-specific links within the Microsoft Teams application to facilitate seamless access to future sessions and study materials.

##### Intervention Safety

The module included practices of moderate intensity, comprising a combination of yoga postures, breathing practices, meditation, and relaxation techniques that were well set out keeping in mind the tele-mode and needs of the participants to avoid any stress or injury. Safety measures were incorporated into the yoga sessions by advising participants to modify postures as needed for ease of practice and stability. Specifically, participants were encouraged to use wall support or support from nearby sturdy furniture when performing standing *asanas* to ensure both comfort and balance. Furthermore, participants were advised to use yoga props such as belts, blocks, and bolsters while performing supine postures as these props provide support while performing *asanas*. The yoga experts and certified instructor from the AHHH hospital provided in-person training to the experienced yoga therapists on the intervention, which led to their comprehensive skill development; adherence to tele-yoga protocols; and, therefore, the safe and standardized implementation of this pioneering tele-yoga trial.

##### Tele-Yoga Platform

The tele-yoga platform was effectively used to engage participants by leveraging a combination of interactive features, accessibility, and personalized support. Real-time internet-based sessions enabled the certified instructor to demonstrate poses, provide feedback, and enhance interaction. The platform ensured compatibility with various devices, including smartphones, tablets, and computers, while offering an intuitive interface facilitating use. Furthermore, automated session reminders and straightforward navigation reduced participation barriers, whereas tools such as attendance monitoring, postsession chats, and progress tracking sustained participant interest. In addition, features such as internet-based forums and shared goals nurtured a sense of community among participants, enhancing their overall experience. The effectiveness of tele-yoga was evidenced by high retention rates, active session participation, and consistently positive feedback, ensuring a seamless experience of the tele-yoga sessions. These elements collectively created an engaging, inclusive environment that successfully replicated many of the benefits of in-person yoga sessions in a remote setting.

#### Waitlist Control Group

The waitlist control group participants in this study, who did not immediately receive the intervention being tested but were placed on a waitlist to receive it after the study’s active phase, were encouraged to maintain their usual lifestyle routines and inform the research team of any alterations in their levothyroxine medication dosage or symptoms, such as fatigue, stress, weight changes, or episodes of depression. These updates were systematically collected during group monthly feedback meetings conducted online using Microsoft Teams, with all data integrated into the final analysis. Throughout the 6-month trial, the waitlist control group continued their usual activities without any intervention, and upon completion of the study, they received the tele-yoga intervention for 6 months, along with video materials.

#### Outcome Measures

##### Primary Measure

The SF-36 is a validated 36-item clinical scale with high test-retest reliability and strong Cronbach α values that assesses physical, psychological, emotional, and social health across various conditions, including thyroid dysfunction [35,36]. The 36 questions are divided into the following eight domains: (1) physical functioning (10 items), (2) role limitation—physical (4 items), (3) bodily pain (2 items), (4) general health perceptions (5 items), (5) energy and vitality (4 items), (6) role limitations—emotional (3 items), (7) social functioning (2 items), and (8) mental health (5 items). The scores were coded and transformed to a scale from 0 to 100 for each domain, where 0 represents the poorest possible health status and 100 indicates the best possible health status. Thus, higher scores correspond to more favorable health outcomes [37].

##### Secondary Measures

To consider other critical measures for the management of hypothyroidism, the following secondary measures were also assessed.

###### Thyroid Profile

This is a standardized test with established validity and reliability for assessing thyroid function that includes measurements of triiodothyronine, thyroxine, and TSH levels [7]. This test offers essential insights into thyroid hormone levels, aiding in the diagnosis of hypothyroidism, assessment of its severity, and evaluation of treatment efficacy.

###### BMI Measure

This is a reliable and validated screening tool used to classify individuals into weight categories, facilitating the assessment of potential health risks [38]. Examining the link between body weight and TSH levels is crucial in hypothyroidism as TSH fluctuations impact metabolism and weight gain, aiding in the targeting of interventions for improved health outcomes. Height (measured via a stadiometer) and weight (measured via an electronic scale) are used to calculate BMI using the following formula: weight (kg)/height^2^ (m^2^) [39].

###### BP Measure

This is a conventional measure with established validity and reliability commonly used to assess both systolic and diastolic pressure through a sphygmomanometer [40]. This measure is vital in hypothyroidism-associated comorbidity as it helps identify hypertension and cardiovascular risks, which are common complications of thyroid dysfunction [41].

###### FAS Measure

This is a validated scale for assessing fatigue, a key hypothyroidism symptom, that aids in evaluating severity and treatment response, with persistent fatigue indicating the need for adjustments or further evaluation [42,43].

###### PSS Measure

This is a 10-item classic stress assessment instrument that is widely recognized for its validity and reliability in measuring stress perception, which helps understand how different situations affect an individual’s feelings and perceived stress levels as high stress impacts thyroid function negatively [44].

###### GIP Scale

This is a reliable and validated 10-item questionnaire that helps understand the personality traits of individuals. This instrument was administered to ascertain the personality types of patients with hypothyroidism as per the *triguna* theory (*sattva* as tranquility or poise, *rajas* as aggression or passion, and *tamas* as ignorance or laziness) [45,46].

#### Monitoring Yoga Performance

The yoga performance assessment (YPA) scale is a validated tool designed to objectively assess the performance and proficiency of individuals practicing yoga. It evaluates multiple dimensions of yoga practice, including postural alignment, breath coordination, stability, flexibility, and overall execution of yoga techniques. The participants were assessed by certified therapists at 2 time points (after 2 months and after 5 months of the intervention) on a scale from 0 to 3 (0=cannot practice at all; 1=needs assistance throughout the practice; 2=needs assistance through some steps of the practice; and 3=can practice with ease without assistance) [47,48].

#### Participant Feedback Procedure

Structured feedback was obtained from participants using a reliable and pretested questionnaire after completion of the 6-month trial conducted on a digital health platform on clarity of the instructions, the effective use of screen positioning, and verbal cues for posture correction, as well as certified instructor and therapist expertise and personalized support [27].

#### Data Collection Method

The clinical scales included the SF-36, FAS, PSS, and GIP, for which questionnaires were prepared in Google Forms and distributed online to participants to complete and submit. The blood samples, along with measurements of height, weight, and BP, were collected in person at the participants’ homes by a reputable laboratory using standardized instruments. All the participants were assessed at 3 distinct time points to evaluate the effect of the tele-yoga intervention on primary and secondary measures: before the intervention (day 0 [–7 days to +7 days]) as time point 1, middle of the intervention (day 90 [–7 days to +7 days]) as time point 2, and after the intervention (day 180 [–7 days to +7 days]) as time point 3. Data analysts received the results of the primary and secondary measures at each time point for secure storage and data privacy until the study’s completion, after which they conducted a comprehensive analysis.

#### Statistical Analysis

The statistical analysis was conducted using SPSS (version 24.0; IBM Corp) and RStudio (version 4.1.2; Posit PBC) to ensure robust and reproducible results. Data preparation adhered to an intention-to-treat approach where missing values were imputed using the multivariate imputation by chained equations model in RStudio. This model ensures that the results reflect the impact of the intervention on the entire sample regardless of participant adherence, helping maintain the integrity of randomization and minimize bias. Hence, replacing missing data based on variable relationships ensured that all 134 participants were included in the analysis maintaining baseline characteristics. Furthermore, the analysis commenced with normality testing of continuous variables using the Shapiro-Wilk test, a method suitable for small to moderate sample sizes due to its sensitivity in detecting deviations from normality. Descriptive statistics were then computed, with continuous variables reported as means and SDs (for normally distributed data) or medians and IQRs (for nonnormally distributed data), whereas categorical variables were summarized as frequencies and percentages.

To evaluate baseline demographic characteristics, between-group differences were analyzed using the chi-square test of independence for categorical variables and the Mann-Whitney *U* test for continuous variables, ensuring appropriate handling of data distributions. Inferential analysis was conducted using a generalized linear model with repeated-measure ANOVA. This model included a within-subject factor assessed across 3 time points and a between-subject factor comparing 2 groups (yoga intervention group vs waitlist control group). Covariates such as age, gender, medications, comorbidities, marital status, educational level, occupation, and duration of illness were adjusted to control for potential confounding effects. The analysis examined group-by-time interaction effects, along with within- and between-subject effects, to understand the intervention’s impact across different time points. Post hoc tests were conducted using the Bonferroni correction to account for multiple comparisons and reduce the risk of type 1 errors. Statistical significance was determined using 2-sided tests, with a threshold of *P*<.05 for significance and *P*<.001 for high significance. The effect size of the intervention on dependent measures was quantified using the partial η^2^, providing insight into the magnitude of the observed effects. This rigorous and systematic approach to statistical analysis ensured transparency and reproducibility, addressing assumptions, handling missing data, and assessing intervention effects comprehensively [49,50].

### Ethical Considerations

This study was approved by the Institutional Ethics Committee of Swami Vivekananda Yoga Anusandhana Samsthana (RES/IEC-SVYASA/222/2022) and was prospectively registered in the Clinical Trials Registry–India (CTRI/2022/03/041047). This study was carried out as per the guidelines and regulations of the World Medical Association Declaration of Helsinki designed for studies of human research [51,52]. The intervention incorporated practices designed to achieve moderate intensity, ensuring accessibility and suitability for a diverse range of participants. Delivered through a telehealth platform, the intervention emphasized safe and controlled movements guided by structured instructions to minimize the risk of improper practice. The selected practices were carefully evaluated for their low potential to cause injury or harm, with specific attention to participant comfort, physical limitations, and ease of adoption in an internet-based setting. This approach ensured a balance between therapeutic benefit and safety, aligning with best practices in remote health interventions. The participants received written and oral information about the study before signing an informed consent form and were told about the option to withdraw their participation from the study at any time, for which they were not required to provide a reason [53]. The privacy and confidentiality of the data, as well as in the collection of informed consent from all participants, were ensured.

## Results

### Participants

**Figure 3:**
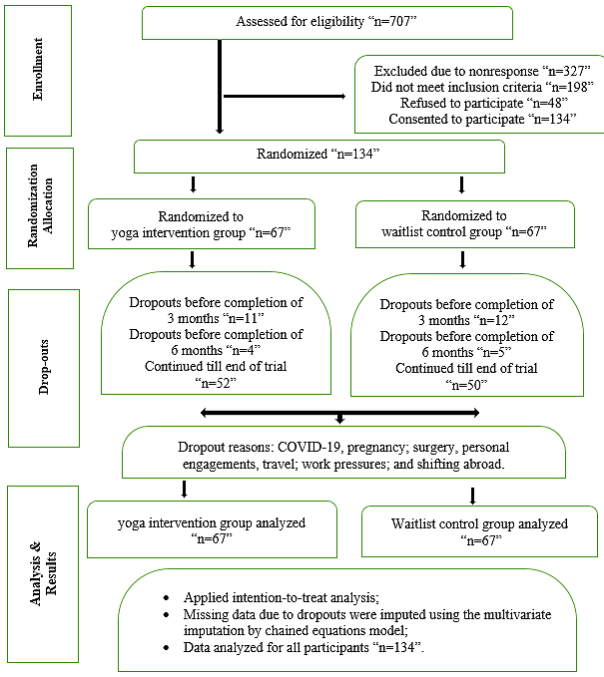
CONSORT (Consolidated Standards of Reporting Trials) flowchart.

Of the 707 patients with hypothyroidism initially contacted, 380 (53.7%) responded and underwent eligibility screening. Of these 380 patients, 182 (47.9%) met the inclusion criteria, with 134 (35.3%) providing informed consent to participate in the trial. These 134 participants confirmed that they had not practiced yoga for at least 3 months before the study, had only a basic understanding of yoga, and expressed interest in learning under supervision, as illustrated in Figure 3.

### Baseline Demographics of the Participants

The cohort of 134 patients with hypothyroidism joined the sessions from different geographical locations in India, including Andhra Pradesh (n=10, 7.5%), Gujarat (n=13, 9.7%), Karnataka (n=32, 23.9%), Madhya Pradesh (n=9, 6.7%), Maharashtra (n=22, 16.4%), New Delhi (n=22, 16.4%), Tamil Nadu (n=15, 11.2%), and West Bengal (n=11, 8.2%). The mean age of the participants was 45.14 (SD 9.15) years, and they underwent and at baseline, participants were assessed for socio-demographic variables, including age, gender distribution, comorbid conditions, marital status, educational level, occupation, duration of illness, and use of levothyroxine medication, along with the evaluation of the primary and secondary measures. The sociodemographic characteristics, as well as the primary and secondary measures, were comparable between the yoga intervention group and waitlist control group groups (*P*>.05), with the exception of levothyroxine dosage, which demonstrated a statistically significant difference (*P*=.03) as the waitlist control group group was observed to have a higher dosage, as detailed in Table 1.

**Table 1:** Baseline demographics of the participants (N=134)^a^.

Variable		Overall	Yoga intervention group (n=67)	Control group (n=67)	*P* value^b^	
Age (y), mean (SD)		45.14 (9.15)	45.01 (8.89)	45.28 (9.46)	.88	
**Gender, n (%)**						.36
	Men	45 (33.6)	25 (37.3)	20 (29.9)		
	Women	89 (66.4)	42 (62.7)	47 (70.1)		
**Comorbidities, n (%)**						>.05
	Diabetes	19 (14.2)	9 (13.4)	10 (14.9)	.80	
	Hypertension	48 (35.8)	27 (40.3)	21 (31.3)	.28	
	Obesity	74 (55.2)	35 (52.2)	39 (58.2)	.48	
	Polycystic ovarian syndrome	68 (50.7)	36 (53.7)	32 (47.8)	.48	
**Marital status, n (%)**						.80
	Married	112 (83.6)	58 (86.6)	54 (80.6)		
	Separated	8 (6)	3 (4.5)	5 (7.5)		
	Single	12 (9)	5 (7.5)	7 (10.4)		
	Widowed	2 (1.5)	1 (1.5)	1 (1.5)		
**Educational level, n (%)**						.07
	Undergraduate	11 (8.2)	3 (4.5)	8 (11.9)		
	Graduate	62 (46.3)	38 (56.7)	24 (35.8)		
	Postgraduate	58 (43.3)	25 (37.3)	33 (49.3)		
	PhD	3 (2.2)	1 (1.5)	2 (3)		
**Occupation, n (%)**						.87
	Business	27 (20.1)	14 (20.9)	13 (19.4)		
	Physician	2 (1.5)	2 (3)	0 (0)		
	Housewife	33 (24.6)	17 (25.4)	16 (23.9)		
	Service worker	63 (47)	28 (41.8)	35 (52.2)		
	Student	5 (3.7)	3 (4.5)	2 (3)		
	Teacher	4 (3)	3 (4.5)	1 (1.5)		
**Duration of illness (y), n (%)**						.34
	0-1	10 (7.5)	4 (6)	6 (9)		
	2-6	26 (19.4)	17 (25.4)	9 (13.4)		
	6-10	56 (41.8)	27 (40.3)	29 (43.3)		
	>10	42 (31.3)	19 (28.4)	23 (34.3)		
**Dosage of medicine (µg), n (%)**						.03
	12.5	1 (0.7)	0 (0)	1 (1.5)		
	25	4 (3)	3 (4.5)	1 (1.5)		
	50	40 (29.9)	27 (40.3)	13 (19.4)		
	75	38 (28.4)	18 (26.9)	20 (29.9)		
	100	51 (38.1)	19 (28.4)	32 (47.8)		
**Primary measure (SF-36^c^), mean (SD)**						>.05
	Physical functioning	43.84 (7.92)	39.63 (8.31)	48.06 (4.60)	.00	
	Role limitation—physical	29.25 (9.49)	29.48 (9.66)	29.03 (9.38)	.06	
	Bodily pain	16.66 (17.22)	14.43 (17.62)	18.90 (16.63)	.68	
	General health perceptions	35.93 (4.91)	35.75 (4.62)	36.12 (5.21)	.97	
	Energy and vitality	45.16 (3.88)	43.91 (4.34)	46.42 (2.90)	.36	
	Role limitation—emotional	45.61 (5.98)	45.15 (6.13)	46.08 (5.84)	.00	
	Social functioning	42.74 (3.88)	42.46 (4.07)	43.02 (3.71)	.58	
	Mental health	49.51 (4.86)	48.88 (4.51)	50.15 (5.18)	.13	
**Secondary measures, mean (SD)**						>.05
	BMI (kg/m^2^)	27.98 (4.78)	27.69 (4.41)	28.27 (5.15)	.88	
	SBP^d^ (mm Hg)	139.62 (11.53)	139.03 (11.91)	140.21 (11.19)	.47	
	DBP^e^ (mm Hg)	90.35 (6.32)	90.61 (6.69)	90.09 (5.97)	.71	
	T3^f^ (pg/mL)	0.95 (0.23)	0.93 (0.22)	0.97 (0.23)	.42	
	T4^g^ (µg/dL)	7.82 (2.03)	7.58 (2.10)	8.06 (1.94)	.22	
	TSH^h^ (mU/L)	5.49 (6.51)	5.07 (3.30)	5.90 (8.62)	,83	
	FAS^i^ (0-50)	42.50 (3.06)	42.46 (3.23)	42.54 (2.91)	.84	
	GIP^j^ (0-30)	15.87 (2.48)	15.91 (2.51)	15.82 (2.47)	.58	
	PSS^k^ (0-40)	31.53 (2.57)	32.01 (2.51)	31.04 (2.56)	.03	

^a^Mann-Whitney *U* test was conducted for continuous data, and the chi-square test was conducted for categorical data.

^b^*P*>.05 indicates similar sociodemographic characteristics; *P*<.05 indicates differences.

^c^SF-36: 36-Item Short Form Health Survey.

^d^SBP: systolic blood pressure.

^e^DBP: diastolic blood pressure.

^f^T3: triiodothyronine.

^g^T4: thyroxine.

^h^TSH: thyroid-stimulating hormone.

^i^FAS: Fatigue Assessment Scale.

^j^GIP: Gita Inventory of Personality.

^k^PSS: Perceived Stress Scale.

### Outcome: Primary Measure

The results for the primary outcomes were derived using repeated-measure ANOVA on the effect of the tele-yoga intervention on the participants before (time point 1), in the middle of (time point 2), and after (time point 3) the intervention on the SF-36 subscales, as shown in Table 2. Interaction between time and group indicated statistically significant effects on all domains of the SF-36 across the time points (*P*<.001). A most significant effect was found in domain 8 (*mental health*; *F*_2, 118_=425.88; η^2^=0.88), followed by domain 5 (*energy and vitality*; *F*_2, 118_=371.73; η^2^=0.86), domain 6 (*role limitations—emotional*; *F*_2, 118_=335.45; η^2^=0.85), domain 1 (*physical functioning*; *F*_2, 118_=316.31; η^2^=0.84), domain 4 (*general health perceptions*; *F*_2, 118_=279.45; η^2^=0.83), domain 7 (*social functioning*; *F*_2, 118_=240.96; η^2^=0.80), domain 2 (*role limitation—physical*; *F*_2, 118_=195.72; η^2^=0.77), and domain 3 (*bodily pain*; *F*_2, 118_=86.41; η^2^=0.59).

**Table 2:** Repeated-measure ANOVA on the effectiveness of the yoga intervention on pre- (time point 1), mid- (time point 2), and postintervention (time point 3) scores on the 36-Item Short Form Health Survey domains^a^.

Variable and time point		Yoga intervention group, mean (SD)	Waitlist control group, mean (SD)	Partial η^2b^	*F* test (*df*; group × time)^c^
**Physical functioning**				0.843	316.31 (2,118)
	1	39.63 (8.31)	48.06 (4.60)		
	2	61.12 (14.32)	40.00 (8.91)		
	3	87.01 (13.95)	36.19. 13.02		
**Role limitation—physical**				0.768	195.72 (2,118)
	1	29.48 (9.65)	29.03 (9.38)		
	2	49.63 (20.17)	19.78 (11.12)		
	3	84.70 (18.44)	11.57 (18.11)		
**Bodily pain**				0.594	86.41 (2,118)
	1	14.43 (17.62)	18.90 (16.64)		
	2	53.23 (20.15)	22.88 (24.07)		
	3	92.03 (15.45)	23.38 (27.83)		
**General health perceptions**				0.826	279.54 (2,118)
	1	35.75 (4,62)	36.12 (5.21)		
	2	65.75 (8.75)	27.76 (11.08)		
	3	78.13 (12.34)	22.31 (15.77)		
**Energy and vitality**				0.863	371.73 (2,118)
	1	43.91 (4.34)	46.42 (2.90)		
	2	51.40 (10.84)	35.13 (12.28)		
	3	83.55 (8.57)	30.30 (14.82)		
**Role limitations—emotional**				0.850	335.45 (2,118)
	1	45.14 (6.13)	46.08 (5.84)		
	2	63.24 (14.41)	38.99 (17.47)		
	3	88.61 (9.41)	25.56 (13.48)		
**Social functioning**				0.803	240.96 (2,118)
	1	42.46 (4.07)	43.02 (3.70)		
	2	51.64 (12.36)	31.23 (9.04)		
	3	84.62 (18.65)	22.91 (14.37)		
**Mental health**				0.878	425.88 (2,118)
	1	48.88 (4.50)	50.15 (5.14)		
	2	72.39 (16.15)	35.67 (12.67)		
	3	88.43 (6.46)	28.06 (14.19)		
**Overall score**				0.930	840.31 (2,118)
	1	299.68 (23.98)	317.78 (26.29)		
	2	468.40 (58.36)	251.45 (54.94)		
	3	687.12 (40.07)	200.28 (91.46)		

^a^Mean scores for each domain closer to 0 indicate the worst possible health, and mean scores closer to 100 indicate the best possible health. Overall scores were calculated as an aggregate of individual domains.

^b^Effect size.

^c^ANOVA test statistic, significant at *P*<.001 (all domains).

### Outcome: Secondary Measures

The results of the secondary outcomes are presented in Table 3. Interaction between time and group showed a statistically significant effect (*P*<.001) on all the secondary variables except thyroxine (*P*<.014). The analysis revealed a significant effect for BMI (*F*_2,118_=49.61; η^2^=0.46), systolic BP (*F*_2,118_=28.92; η^2^=0.33), diastolic BP (*F*_2,118_=23.99; η^2^=0.28), triiodothyronine (*F*_2,118_=14.47; η^2^=0.19), TSH (*F*_2,118_=24.80; η^2^=0.29), FAS (*F*_2,118_=315.81; η^2^=0.84), GIP (*F*_2,118_=350.71; η^2^=0.85), and PSS (*F*_2,118_=1094.16; η^2^=0.94) with *P*<.001 and for thyroxine (*F*_2,118_=5.17; η^2^=0.08).

**Table 3:** Repeated-measure ANOVA on the effectiveness of the yoga intervention across time points (before [time point 1], in the middle of [time point 2], and after [time point 3] the intervention) on the secondary measures^a^.

Variable and time point			Yoga intervention group, mean (SD)		Waitlist control group, mean (SD)		Partial η^2b^			*F* test (*df*; group × time)^c^	
**BMI (kg/m^2^)**								0.46			49.61 (2, 118)
	1	27.69 (4.41)		28.27 (5.15)							
	2	27.33 (4.30)		28.96 (5.16)							
	3	24.68 (3.49)		28.86 (5.00)							
**SBP^d^ (mm Hg)**								0.33			28.92 (2, 118)
	1	139.03 (11.90)		140.21 (11.19)							
	2	135.60 (11.59)		142.81 (10.71)							
	3	132.79 (10.54)		143.72 (10.46)							
**DBP^e^ (mm Hg)**								0.28			23.99 (2, 118)
	1	90.61 (6.69)		90.09 (5.97)							
	2	88.30 (5.79)		89.81 (5.59)							
	3	85.87 (5.51)		91.60 (5.44)							
**T3^f^ (pg/mL)**								0.19			14.47 (2, 118)
	1	0.93 (0.22)		0.97 (0.23)							
	2	0.96 (0.22)		0.90 (0.24)							
	3	1.06 (0.28)		0.88 (0.19)							
**T4^g^ (µg/dL)**								0.08			5.17 (2, 118)
	1	7.58 (2.10)		8.06 (1.94)							
	2	8.28 (2.49)		7.87 (1.92)							
	3	9.60 (3.70)		8.32 (2.62)							
**TSH^h^ (mU/L)**								0.29			24.80 (2, 118)
	1	5.07 (3.30)		5.91 (8.62)							
	2	3.65 (2.98)		8.08 (13.56)							
	3	3.14 (2.47)		8.14 (3.97)							
**FAS^i^ (0-50)**								0.84			315.81 (2, 118)
	1	42.46 (3.23)		42.54 (2.91)							
	2	24.19 (4.02)		37.06 (2.44)							
	3	18.10 (3.68)		39.84 (3.82)							
**GIP^j^ (0-30)**								0.85			350.71 (2, 118)
	1	15.91 (2.51)		15.82 (2.47)							
	2	24.70 (2.43)		17.51 (2.59)							
	3	26.69 (1.81)		15.40 (1.81)							
**PSS^k^ (0-40)**								0.95			1094.16 (2, 118)
	1	32.01 (2.51)		31.04 (2.55)							
	2	21.81 (5.12)		31.16 (3.17)							
	3	9.58 (1.54)		32.87 (3.66)							

^a^The optimum range values for a healthy level across parameters are as follows: 19 to 25 kg/m^2^ for BMI, 120 to 130 mm Hg for systolic blood pressure, 80 to 85 mm Hg for diastolic blood pressure, 5 to 12 µg/dL for thyroxine, 2.3 to 4.1 pg/mL for triiodothyronine, and 0.45 to 4.12 mU/L for thyroid-stimulating hormone.

^b^Effect size.

^c^ANOVA test statistic; significance at *P*<.001 for all variables and at *P*<.05 for thyroxine.

^d^SBP: systolic blood pressure.

^e^DBP: diastolic blood pressure.

^f^T3: triiodothyronine.

^g^T4: thyroxine.

^h^TSH: thyroid-stimulating hormone.

^i^FAS: Fatigue Assessment Scale (higher scores indicate higher levels of fatigue).

^j^GIP: Gita Inventory of Personality (higher scores indicate a more positive personality profile).

^k^PSS: Perceived Stress Scale (higher scores indicate higher levels of perceived stress).

### Adherence Assessment

Of the 134 participants, 1:1 randomization assigned 67 participants each to the yoga intervention group and waitlist control group, out of which 52 (78%) participants in the yoga intervention group and 50 (75%) in the waitlist control group completed the program, resulting in a total attrition rate of 23.9% (32/134). Overall, 102 (76.1%) participants adhered to the program across both groups. The average attendance rate was 83% (SD 10.2%) in the yoga intervention group for the supervised online sessions, with participants attending at least 120 out of 144 of the scheduled treatment sessions. Additionally, 8% (SD 1.36%) reported compensating for missed sessions by practicing at home using the provided video of the scientific yoga module. These home sessions were tracked through a separate attendance log, which was updated after verification of the submitted photos or videos.

### YPA Scale

The results of the YPA scale used to evaluate participants’ overall performance in each yoga posture at 2 time points showed significant improvement, with average scores increasing from 65.08 (SD 10.97) to 88.62 (SD 11.18; paired-sample 2-tailed *t* test; *P*<.001), confirming that most participants could perform scientific yoga module practices with ease.

### Participant Feedback Assessment

The results of the structured feedback obtained from participants using a pretested questionnaire [27] showed participant satisfaction, with scores for (1) regularity and ease of practice (defined as the consistency and simplicity with which participants integrated and performed scientific yoga module practices in their daily routine; 64/67, 95%), (2) efficacy of the module (defined as effectiveness in achieving desired outcomes for the participants; 64/67, 95%), (3) construct satisfaction (defined as the degree to which the design, structure, and content met participants’ expectations; 63/67, 94%), and (4) adverse effects (defined as injuries, discomfort, or increased stress; apart from a few participants who indicated muscular pain in the first week of the intervention, which was resolved within a week of practice; 64/67, 95% of the participants reported “No adverse effects”), as shown in Table 4.

**Table 4:** Tele–scientific yoga module (SYM) feedback analysis (N=67)^a^.

Description			Yes, n (%)		No, n (%)		Sometimes, n (%)
**Regularity and ease of practice**							
	“Was the tele-yoga easy to practice through online coaching mode?”	65 (97.5)		0		2 (2.5)	
	“Did you find tele-yoga sessions easy to integrate into your daily routine?”	63 (94.5)		0		4 (5.5)	
	“Did you feel motivated to continue the practice using the SYM video during home practice?”	62 (93.5)		0		5 (6.5)	
	“Were you able to easily join the online sessions using the link provided?”	65 (97.5)		0		2 (2.5)	
	“Were the instructions for each yoga pose clear and easy to understand through tele-mode?”	66 (98.5)		0		1 (1.5)	
	“Did the screen positioning help to follow the poses during the online sessions?”	66 (98.5)		0		1 (1.5)	
	“Were the verbal cues provided by the instructor during the session helpful in correcting your posture?”	63 (94.5)		0		4 (5.5)	
	“Did the instructor demonstrate expertise in guiding the tele-yoga sessions?”	63 (94.5)		0		4 (5.5)	
	“Did the therapist provide sufficient individualized support to improve your practices?”	62 (93.5)		0		5 (6.5)	
	“Did you feel confident performing the yoga poses with the guidance and support provided during online sessions?”	63 (94.5)		0		4 (5.5)	
**Efficacy of the module**							
	“Was the tele-yoga suitable for the age group between 18 to 60 years?”	62 (93.5)		0		5 (6.5)	
	“Was the tele-yoga appropriate for both genders (Male and Female)?”	63 (94.5)		0		4 (5.5)	
	“Can the SYM be considered as a moderate-intensity practice?”	66 (98.5)		0		1 (1.5)	
	“Can the SYM be practiced without supervision at home using the video?”	62 (93.5)		0		5 (6.5)	
	“Was the SYM safe to practice at home?”	63 (94.5)		0		4 (5.5)	
	“Do you think the SYM is suitable for treating hypothyroidism effectively and improving overall well-being?”	66 (98.5)		0		1 (1.5)	
**Construct satisfaction**							
	“Were you satisfied with the sequence of the yoga practices in the tele-yoga sessions?”	66 (98.5)		0		1 (1.5)	
	“Did the flow of poses during each session feel coherent and well-structured?”	63 (94.5)		0		4 (5.5)	
	“Were the practices appropriately paced for your comfort and skill level?”	62 (93.5)		0		5 (5.5)	
	“Did the session structure help you stay engaged and focused throughout?”	62 (93.5)		0		5 (6.5)	
	“Were the warm-up and cool-down segments effectively integrated into the sessions?”	64 (95.5)		0		3 (4.5)	
	“Did you find the balance between physical poses and relaxation techniques satisfactory?”	60 (89.5)		0		7 (10.5)	
	“Was the progression of poses from session to session logical and beneficial?”	61 (91.0)		0		6 (9.0)	
	“Did the tele-yoga practices meet your expectations in terms of structure and content?”	66 (98.5)		0		1 (1.5)	
**Adverse effects**							
	“Did you encounter any adverse events or discomfort practicing tele-yoga?”	1 (1.0)		64 (95.5)		2 (3.5)	

^a^Total average scores were (1) 64/67, 95% for regularity and ease of practice, (2) 64/67, 95% for efficacy of the module, (3) 63/67, 94% for construct satisfaction, and (4) 64/67, 95% for No adverse effects. Overall satisfaction with the tele-SYM was reported at 95.05% (results close to 100 denote greater satisfaction).

## Discussion

### Principal Findings

The findings of this study indicate that the yoga intervention group demonstrated significantly greater improvement than the waitlist control group across both primary and secondary measures after the intervention (*P*<.001). The scientific yoga module’s integration into digital health pathways highlights its potential as a holistic component for thyroid care. Clinically, participants demonstrated significant improvement in the primary measure of health-related QoL, with enhanced physical and psychosocial functioning. Secondary measures also showed meaningful gains, including reductions in stress and improvements in self-awareness, BMI, and thyroid hormone levels, suggesting better metabolic regulation, weight management, and overall well-being. These findings highlight the efficacy of the scientific yoga module delivered as an eHealth intervention for patients with hypothyroidism and support its use as an adjunctive tele-yoga therapy, helping alleviate common symptoms such as fatigue, musculoskeletal pain, weight gain, and cognitive challenges that are often insufficiently addressed through conventional medication alone [54-56]. Tele-yoga literature emphasizes that participants value the accessibility of eHealth technologies, allowing them to attend sessions from home, avoid travel costs, and integrate classes into their schedules [57]. This study confirms the effectiveness of the scientific yoga module as an eHealth intervention for thyroid health, supporting existing literature on tele-yoga’s role in ensuring care continuity. This study provides a viable home-based alternative for managing hypothyroid symptoms and contributes to the literature by highlighting the sustainability of remote treatment and promoting a global wellness community [58].

### Theoretical Contribution and Comparison to Prior Work

This study makes several significant contributions to the literature and collectively provides valuable insights into the utility of digital health solutions, specifically tele-yoga–based interventions, in addressing multidimensional health needs in patients with hypothyroidism, from stress and metabolic regulation to reproductive and gender-specific health considerations. Studies have indicated that hypothyroidism is frequently overlooked due to its often asymptomatic nature and, if left untreated, can result in comorbidities such as obesity and cardiovascular diseases [59]. First, this study advances theoretical understanding by positioning the tele-scientific yoga module as an effective tool for managing hypothyroidism along with supporting stress reduction, metabolic health, and overall well-being, highlighting the potential of digital platforms to reach diverse and geographically dispersed populations. This study addresses the need for rigorous scientific investigation on tele-yoga, as highlighted in a recent scoping review [60]. It contributes through transparent reporting, timely publication of the protocol and feasibility study, expert instruction with real-time guidance, appropriate intervention duration, detailed descriptions of yoga practices (dosage and frequency), and high participant adherence, advancing evidence on yoga’s role in managing hypothyroidism. Notably, this study reinforces digital health’s potential for hypothyroidism management by exploring synchronous tele-yoga delivery alongside telehealth’s established clinical effectiveness and provides a structured, methodologically sound approach, evaluating outcomes over 6 months with a diverse participant pool [61,62].

In addition, this work supports previous evidence suggesting yoga’s benefits as a lifestyle therapy for improving metabolic health, as highlighted in the meta-analysis by Chu et al [63]. This meta-analysis highlighted the benefits of tele-yoga in managing obesity among patients with hypothyroidism, particularly in cases in which conventional pharmacological treatments may be insufficient, thereby substantiating tele-yoga’s role as a holistic approach that complements standard therapies [64]. This finding is consistent with those of previous studies on yoga’s positive effects in managing weight fluctuations and supports the broader use of eHealth in metabolic health promotion [65]. In addition, this work reinforces the association between hypothyroidism and reproductive health challenges, specifically infertility in women. Effective lifestyle management through tele-yoga interventions has been shown to support improved QoL for participants and is particularly beneficial in managing hypothyroidism-related reproductive issues [66]. Moreover, psychological declines observed in middle-aged women in the waitlist control group align with studies indicating midlife as a vulnerable period for emotional disturbances, emphasizing tele-yoga’s supportive role in this demographic [67]. This work highlights yoga’s effectiveness in addressing hormonal imbalances by potentially impacting the hypothalamic-pituitary-ovarian axis, which is often disrupted in patients with hypothyroidism. These findings further expand the scope of digital health in chronic disease management.

Furthermore, this study allowed for more reliable assessments of the scientific yoga module’s impact as an eHealth intervention for patients with hypothyroidism as it examined a large sample size compared to previous studies with smaller cohorts, thus enhancing the generalizability and replicability of the results in this domain [68,69]. Moreover, compared to previous tele-yoga trials, this study demonstrated significant participant attendance improvement, achieving an attendance rate of 83% (SD 10.2%) and an overall dropout of 23.9% (32/134) in both yoga intervention group and waitlist control group over the 6-month trial period, confirming the successful administration of the intervention [64,69]. This study meticulously ensured that the practices of the scientific yoga module were thoughtfully designed, with moderate intensity and adaptable practices, making it accessible and suitable for participants across various age groups and both genders, thus reducing physical and psychological barriers to participation. The presence of a skilled tele-yoga instructor facilitated effective, personalized sessions, fostering participant confidence and engagement. Real-time, individualized guidance from a trained therapist enhanced the experience by addressing concerns and providing modifications. Thus, as compared to previous tele-yoga interventions, the combined elements leveraged in this study created a supportive and adaptable environment, promoting consistent participation and minimizing dropout rates.

In addition, this study revealed distinct gender-related trends in comorbidities associated with hypothyroidism, such as obesity and hypertension. In female individuals, obesity frequently co-occurred with polycystic ovarian syndrome beginning in early adulthood, suggesting a relationship between hormonal function and reproductive health [70-72]. Among male individuals, hypertension was more prevalent, particularly after the age of 50 years, possibly due to delayed symptom recognition and diagnosis [73]. Notably, this work observed an increase in hypothyroidism prevalence among men compared to previous research, which calls for further investigation into evolving gender prevalence trends and associated comorbidities using eHealth research—an interesting avenue for further research [74]. Moreover, the observed reduction in stress levels in the yoga intervention group compared to the waitlist control group aligns with previous research that demonstrates yoga’s capacity to activate the autonomic nervous system by enhancing parasympathetic activity [75], effectively reducing stress and affirming tele-yoga’s role in managing psychological symptoms associated with hypothyroidism. These findings further establish tele-yoga as a viable eHealth intervention for stress management in patients with hypothyroidism, highlighting its potential for enhancing well-being in a remote health care context [76-78]. These insights confirm the scientific yoga module to be safe, feasible, and acceptable as a telehealth intervention, demonstrating the potential of digital platforms to deliver consistent, accessible, and user-centered therapeutic yoga aligned with remote health care standards.

### Strengths

This study introduced a novel tele-yoga module that integrates ancient yoga with modern medicine in a telehealth framework, offering a holistic, evidence-based approach to managing hypothyroidism. This study demonstrated significant improvements in health-related QoL and symptom management, underscoring the therapeutic potential of tele-yoga as a complementary treatment for hypothyroidism. High adherence and low dropout rates over 6 months confirm the feasibility and user-friendliness of the digital intervention, particularly for individuals with limited access to in-person care. The tele-yoga module’s flexibility across age, gender, and lifestyles addresses barriers such as distance, cost, time, and limited professional availability, offering a scalable, sustainable model for chronic condition management. These findings support tele-yoga as an innovative, patient-centered telehealth strategy that integrates traditional wellness with modern technology to enhance outcomes in hypothyroidism.

### Limitations

Some limitations of this study that can be examined further and, thus, offer avenues for future research include the following. First, there is a potential risk of motivational bias as participants were exclusively recruited from the AHHH patient registry, where yoga is an established treatment modality. This preexisting knowledge and acceptance of yoga might drive a favorable predisposition to the intervention, influencing participants’ responses. Second, blinding was not possible for either participants or instructors, which could have affected participants’ performance and instructors’ teaching style due to existing familiarity. This limitation, while inherent in behavioral and telehealth interventions, highlights the need for innovative methodologies to minimize bias in nonpharmacological studies.

### Future Scope

For future research in this domain, it is recommended that, in addition to evaluating thyroid profile, the inclusion of a specific biomarker (thyroid peroxidase antibody) be considered as it is a critical indicator of autoimmune thyroid disorders, which would provide deeper insights into the underlying autoimmune mechanisms contributing to hypothyroidism. Furthermore, a longitudinal study using the scientific yoga module within a telehealth framework and regular follow-ups over an extended period of >2 years would allow researchers to evaluate the sustained benefits of yoga practice for patients with hypothyroidism. Preliminary discussions are underway to design a longitudinal study incorporating the scientific yoga module within a telehealth framework with follow-ups over 2 years and a focus on global applicability. This study aims to evaluate the sustained benefits of yoga practice and the long-term impact of the tele-yoga module on QoL, enhancing its potential as a therapeutic intervention for hypothyroidism. Global research expansion would improve generalizability and provide insights into the intervention’s adaptability, accessibility, and effectiveness across diverse cultural and demographic contexts.

### Conclusions

This trial is the first to demonstrate tele-yoga’s benefits as an adjunct therapy for patients with hypothyroidism on levothyroxine, with the potential for dose reduction and QoL improvement. It offers a cost-effective, accessible platform, overcoming barriers associated with center-based programs, supporting sustained practice and overall well-being. This trial provides health care researchers and practitioners with information about using simple and effective mind-body therapy as an adjunct tool for both the prevention and treatment of hypothyroidism using the telehealth model.

## Data Availability

All data generated or analyzed during this study are stored electronically in the database of the Arogyadhama Holistic Health Home, Bengaluru, India, and can be made available upon reasonable request from author AS.

## Appendix

Multimedia Appendix 1 Scientific yoga module for hypothyroidism designed for digital delivery.

Multimedia Appendix 2 CONSORT-eHEALTH checklist (V 1.6.1).

## Abbreviations

AHHH: Arogyadhama Holistic Health Home
BP: blood pressure
CONSORT: Consolidated Standards of Reporting Trials
FAS: Fatigue Assessment Scale
GIP: Gita Inventory of Personality
PSS: Perceived Stress Scale
QoL: quality of life
RCT: randomized controlled trial
SF-36: 36-Item Short Form Health Survey
TSH: thyroid-stimulating hormone
YPA: yoga performance assessment
