# Supplementary material for: Enhancing Quality of Life in Patients With Hypothyroidism Using a Scientific Yoga Module: Randomized Controlled Trial
Source: J Med Internet Res. 2025 Jun 26;27:e54078. doi: 10.2196/54078 (PMC12246764; doi:10.2196/54078)
Supplement: Multimedia Appendix 1 [file jmir_v27i1e54078_app1.docx]

Scientific yoga module for hypothyroidism designed for digital delivery.

| Yoga practice | | Time (min) | CVR^a^ | Benefits | Postures |
| --- | --- | --- | --- | --- | --- |
| **Standing asanas (8 min)** | | | | | |
|  | Grieva shakti (neck movements—front-back-side bending and twisting and rotation) | 2 | 0.85 | Strengthens the neck and activates the thyroid gland | 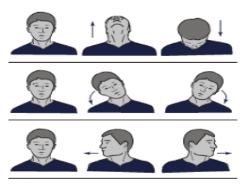 |
|  | Ardha-chakrasana (half-wheel posture) | 2 | 0.95 | Works on the throat and neck region; promotes a healthy metabolic function | 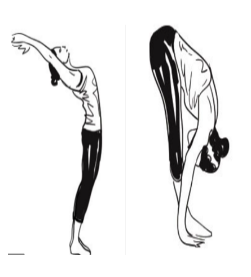 |
|  | Padahastasan (hand-to-toe posture) | 2 | 0.50 | Works on the throat and neck region; promotes a healthy ametabolic function | (—) |
|  | Sequential (ardha-chakrasana and padahastasana) | 2 | 0.42 | Works on the throat and neck region; promotes a healthy metabolic function | (—) |
| **Sitting asanas (8 min)** | | | | | |
|  | Ushtrasana (camel posture) | 2 | 1.00 | It stretches the glands on the neck and strengthens the shoulder and thigh muscles | 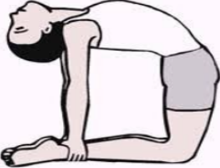 |
|  | Sahankasana (rabbit posture) | 2 | 0.55 | Improves blood flow toward the neck and head region and provides relaxation | 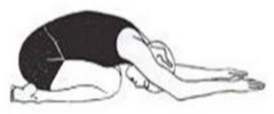 |
|  | Ardha-matsendrasana (half-fish posture) | 4 | 0.45 | A self-complementary deep twisting posture that helps with proper functioning at the gland level | 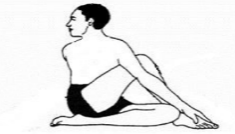 |
| **Prone asanas (8 min)** | | | | | |
|  | Bhujangasana (cobra posture) | 2 | 0.90 | Massages the glands and helps overcome lethargy as it has an effect on the solar plexus at the navel and on the throat region | 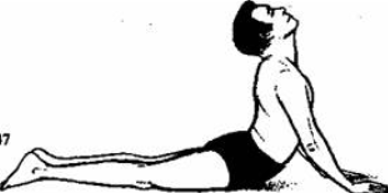 |
|  | Dhanurasana (bow posture) | 2 | 0.80 | Stretches the thyroid gland and compels it to produce the required amount of thyroid hormone for regulating metabolism | 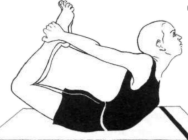 |
|  | Sequential (parvatasana and bhujangasana stretch) | 2 | 0.50 | Tones the spinal nerves and balances the nervous system | 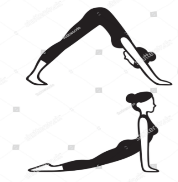 |
|  | Makrasana (crocodile posture) | 2 | 0.35 | Complete relaxation in prone position | 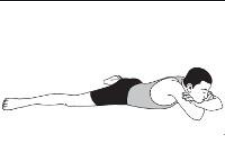 |
| **Supine asanas (10 min)** | | | | | |
|  | Vipareeta karni sarvangasana (supported inverted posture) | 2 | 0.90 | Exerting pressure on the thyroid gland helps in improving blood circulation and nourishes the thyroid gland | 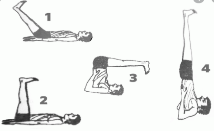 |
|  | Halasana (plow posture) | 2 | 0.70 | By squeezing the thyroid gland, the stagnant secretion is released | 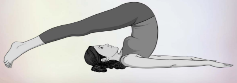 |
|  | Sequential (pashchimotanasana and halasana) | 4 | 0.50 | These supine postures provide great stretch and squeeze to the thyroid and adrenal glands for proper secretion of thyroid hormone and cortisol to combat stress | 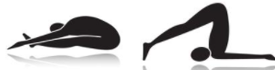 |
|  | Matsyasana (fish posture) | 2 | 0.90 | Stretching the front of the neck fully and placing the crown on the floor activates the pituitary and thyroid glands | 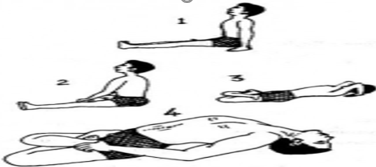 |
| **Suryanamaskar (12 min)** | | | | | |
|  | Surya namaskar (sun salutation) | 11 | 0.88 | A sequence of gracefully linked asanas that are synchronized with breathing that enhances metabolic efficiency and enables overall health and well-being | 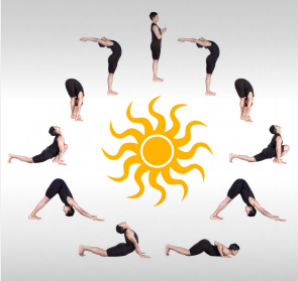 |
|  | Shavasana (corpse position) | 1 | 0.90 | A relaxation that harmonizes the energies of the mind-body system | 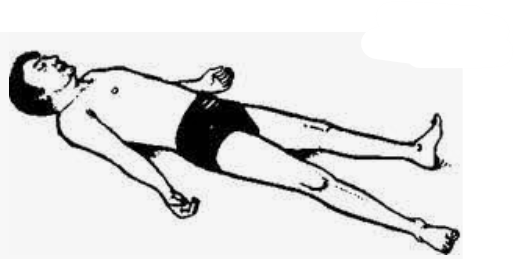 |
| **Mudras-bandas pranayama (12 min)** | | | | | |
|  | Simha mudra (lion gesture) | 2 | 0.95 | It improves thyroid health | 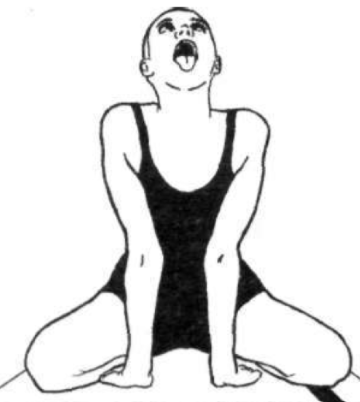 |
|  | Jalandhara banda (throat locking) | 2 | 0.70 | It awakens the inner energy centers, especially the vishuddhi chakra, and regulates thyroid function | 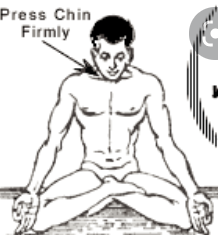 |
|  | Ujjaii pranayama (victorious breath) | 2 | 0.80 | Activates the thyroid gland and regulates the secretion of hormones | 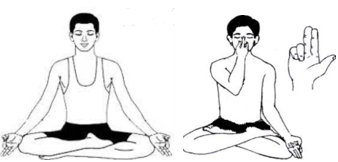 |
|  | Brahmri pranayama (bumblebee breath) | 2 | 0.50 | Calms the mind and has significant benefits for the overall endocrine system | 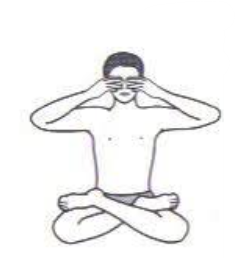 |
|  | Nadi shodhana pranayama (balancing of breath) | 2 | 0.65 | Activates the nadi system (psychic channels), brings better balance to the nervous system, and helps combat stress | 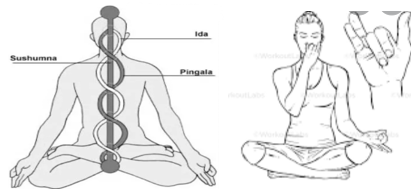 |
|  | A-kara with shanka mudra (conch bell with a-kara) | 2 | 0.45 | A-kara sound from the throat by adopting shanka mudra activates the vishuddhi chakra and helps regularize thyroid hormone secretion | 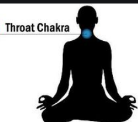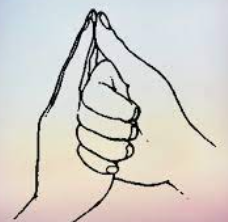 |
|  | Shavasana (corpse position) | 2 | 0.55 | Brings complete calmness and mind-body relaxation | 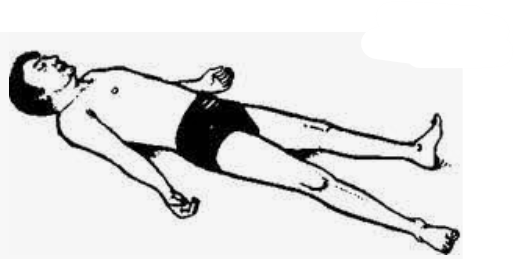 |
| ^a^CVR: content validity ratio | | | | | |

.
